# Traditional Japanese Medicine Daikenchuto Improves Functional Constipation in Poststroke Patients

**DOI:** 10.1155/2014/231258

**Published:** 2014-06-25

**Authors:** Takehiro Numata, Shin Takayama, Muneshige Tobita, Shuichi Ishida, Dai Katayose, Mitsutoshi Shinkawa, Takashi Oikawa, Takanori Aonuma, Soichiro Kaneko, Junichi Tanaka, Seiki Kanemura, Koh Iwasaki, Tadashi Ishii, Nobuo Yaegashi

**Affiliations:** ^1^Department of Obstetrics and Gynecology, Tohoku University Graduate School of Medicine, 2-1 Seiryo-Machi, Aoba Ward, Sendai City, Miyagi 980-8575, Japan; ^2^Department of Kampo Medicine, Tohoku University Hospital, 1-1 Seiryo-Machi, Aoba Ward, Sendai City, Miyagi 980-8574, Japan; ^3^Comprehensive Education Center for Community Medicine, Tohoku University Graduate School of Medicine, 2-1 Seiryo-Machi, Aoba Ward, Sendai City, Miyagi 980-8575, Japan; ^4^National Yonezawa Hospital, 26100-1 Oh-Aza Misawa, Yonezawa City, Yamagata 992-1202, Japan; ^5^Ishinomaki Rehabilitation Hospital, 1-2-21 Kadonowaki-cho, Ishinomaki City, Miyagi 986-0834, Japan; ^6^Miyagi Rifu Ekisaikai Hospital, 51 Morigo Aza Shintaishido, Rifu Town, Miyagi 981-0103, Japan; ^7^Hikarigaoka Spellman Hospital, 6-7-1 Higashi-Sendai, Miyagino Ward, Sendai City, Miyagi 983-0833, Japan; ^8^National Hachinohe Hospital, 3-13-1 Fukiage, Hachinohe City, Aomori 031-0003, Japan; ^9^Wakuya Medical and Welfare Center, 278 Wakuya Aza Nakakonan, Wakuya Town, Miyagi 987-0121, Japan; ^10^Department of Education and Support for Community Medicine, Tohoku University Hospital, 1-1 Seiryo-Machi, Aoba Ward, Sendai City, Miyagi 980-8574, Japan; ^11^Center for Traditional Asian Medicine and Home Healthcare, Southern Tohoku General Hospital, 1-2-5 Satonomori, Iwanuma City, Miyagi 989-2483, Japan

## Abstract

Poststroke patients with functional constipation, assessed by the Rome III criteria, from 6 hospitals were recruited in a study on the effects of the traditional Japanese medicine Daikenchuto (DKT) on constipation. Thirty-four patients (17 men and 17 women; mean age: 78.1 ± 11.6 years) were randomly assigned to 2 groups; all patients received conventional therapy for constipation, and patients in the DKT group received 15 g/day of DKT for 4 weeks. Constipation scoring system (CSS) points and the gas volume score (GVS) (the measure of the intestinal gas volume calculated from plain abdominal radiographs) were recorded before and after a 4-week observation period. The total score on the CSS improved significantly in the DKT group compared to the control (*P* < 0.01). In addition, scores for some CSS subcategories (frequency of bowel movements, feeling of incomplete evacuation, and need for enema/disimpaction) significantly improved in the DKT group (*P* < 0.01, *P* = 0.049, and *P* = 0.03, resp.). The GVS was also significantly reduced in the DKT group compared to the control (*P* = 0.03). DKT in addition to conventional therapy is effective in treating functional constipation in poststroke patients. This study was a randomized controlled trial and was registered in the UMIN Clinical Trial Registry (no. UMIN000007393).

## 1. Introduction

There were over 1.34 million cerebrovascular patients in 2008 reported by the Japanese Ministry of Health, Labour, and Welfare [1]. Constipation is one of the complications seen in poststroke patients. Stratified by stroke severity on the National Institutes of Health Stroke Scale, the reported incidence of constipation in poststroke patients is 38.9% to 88.2% [2]. Functional constipation is thought to originate from decreased gastrointestinal motility as well as from decreased autonomic nervous system efficiency, impaired physical activity, abdominal muscle weakness secondary to hemiplegia, and diet [3]. Conventional therapy to control constipation involves the use of laxatives or stimulant purgatives, and these drugs are often used in the long term in chronic constipation patients [4]. However, patients can develop a tolerance to laxatives or stimulant purgatives, and paralytic ileus occasionally occurs in the clinical setting, even with conventional therapy [5].

DKT has historically been used to treat gastrointestinal dysfunction with abdominal coldness and pain in many East Asian countries, including Japan and China [6]. Recently, it has also been used to prevent ileus after gastrointestinal surgery and to treat irritable bowel syndrome [7]. Horiuchi et al. reported that DKT significantly improved abdominal bloating and pain and reduced intestinal gas volume in patients with intractable functional constipation [8]. Physiological reactions to the administration of DKT have been reported as promoting gastrointestinal motility [9–13] and increasing intestinal blood perfusion [14–19]. DKT's effectiveness in treating defecation disorders in patients with cerebrovascular disease is commonly observed in the clinical setting. Potential mechanisms underlying the physiological responses to DKT have been investigated in animal models and include elevated levels of plasma vasoactive intestinal polypeptide [14, 17, 20], substance P [14, 17, 21, 22], motilin [23–25], and acetylcholine [10, 11, 13, 26–28], which promote gastrointestinal motility, as well as calcitonin gene-related peptide (CGRP) [14, 15, 17, 21] and adrenomedullin [15, 16, 29, 30], which increase intestinal blood flow. Poststroke patients are at risk for arteriosclerosis and often experience abdominal pain accompanied by a cold sensation in the abdomen associated with low blood perfusion in the mesenteric arteries. DKT has been used to treat defecation disorders with abdominal coldness and pain caused by decreased intestinal motility and blood flow. We previously reported that administration of DKT increased blood flow in the superior mesenteric artery and promoted intestinal peristalsis in healthy subjects [18, 19]. Sato et al. reported that DKT significantly increased plasma CGRP levels in healthy subjects [21]. Therefore, plasma CGRP may be a useful biomarker to evaluate the effects of DKT on intestinal blood flow.

This study aimed to investigate the efficacy of DKT in treating functional constipation in poststroke patients. In addition, this study investigated the impact of DKT therapy on CGRP concentration.

## 2. Methods

### 2.1. Subject Eligibility Criteria

Eligible patients were aged 20 to 99 years of both genders, had been diagnosed with functional constipation according to the Rome III criteria [31], and remained stable over a 6-month period from the onset of cerebral hemorrhage, cerebral infarction, and subarachnoid hemorrhage. Patients received nutrition orally or through a nasogastric or gastrostomy tube. Patients with concurrent diabetes were required to have an HbA1c (NGSP) less than 9%.

### 2.2. Subject Exclusion Criteria

Patients meeting or diagnosed with any of the following criteria were excluded: risk of intestinal adhesion following abdominal surgery, inflammatory bowel disease, or malignant gastrointestinal disease; hypoxic encephalopathy or myelopathy; history of interstitial pneumonia; liver and/or kidney dysfunction; cancer; and neurodegenerative disease, such as Parkinson's disease or spinocerebellar degeneration. However, patients who underwent laparoscopic cholecystectomy or underwent percutaneous endoscopic gastrostomy were not excluded because the invasiveness of the operation was minimal.

### 2.3. Patient Recruitment

From September 2012 to December 2013, eligible subjects were recruited from 6 hospitals: National Yonezawa Hospital, Ishinomaki Rehabilitation Hospital, National Hachinohe Hospital, Hikarigaoka Spellman Hospital, Miyagi Rifu Ekisaikai Hospital, and Wakuya Medical and Welfare Center.

### 2.4. Logistics

Subjects were randomly assigned to the DKT group or the control group. The study protocol was conducted in accordance with the Declaration of Helsinki and was approved by the Institutional Review Boards of Tohoku University Hospital and the 6 collaborating hospitals. Written informed consent was obtained from all patients or their families.

### 2.5. Trial Methods

The study protocol included an intention to treat analysis. The control group underwent conventional therapy for constipation, such as laxative administration, enemas, and disimpaction. In addition to conventional therapy, the DKT group continuously received 5.0 g of Daikenchuto extract granules (TJ-100, Tsumura & Co., Tokyo, Japan) 3 times a day before meals for 4 weeks. Each clinical parameter was measured before and after the 4-week trial. Fifteen grams of TJ-100 (DKT) extract granules contains a dried herbal extract mixture in the following proportions: Ginseng radix (Araliaceae,* Panax ginseng* C.A. Meyer, Radix) (3.0 g), processed ginger root (Zingiberaceae,* Zingiber officinale* Roscoe, rhizoma) (5.0 g),* Zanthoxylum* fruit (Rutaceae,* Zanthoxylum piperitum* De Candolle, pericarpium) (2.0 g), and saccharum granorum (the candy produced from maltose) (10.0 g). This formulation is registered in the Japanese Pharmacopoeia Sixteenth Edition [32]. The production and supply processes for TJ-100 comply with good manufacturing practice standards for Kampo products and have been approved by the Japanese Ministry of Health, Labour, and Welfare.

### 2.6. Evaluation of Clinical Symptoms

#### 2.6.1. Activities of Daily Living

The Barthel Index was recorded for each patient at study enrollment to assess activities of daily living [33].

#### 2.6.2. Clinical Constipation Scores

Clinical scores for constipation were recorded before and after the 4-week trial period using the constipation scoring system (CSS, see the appendix) [34]. Questionnaires concerning constipation were administered to patients; however, if the patients could not completely answer the question, their families or nurses evaluated the questions depending on the objective findings (i.e., painful evacuation effort or abdominal pain before defecation was evaluated by family members or nurses using the patients' facial expressions; feeling of incomplete evacuation was evaluated with abdominal fullness after defecation). Because it was difficult to evaluate Q5 (“Time: minutes in lavatory per attempt”) in the CSS for bedridden subjects using diapers, we removed Q5 from the statistical analysis. Evaluations before and after the administration of DKT were performed by the same family member or nurse with blinding of DKT administration.

#### 2.6.3. Plain Abdominal Radiography

**Figure 1:**
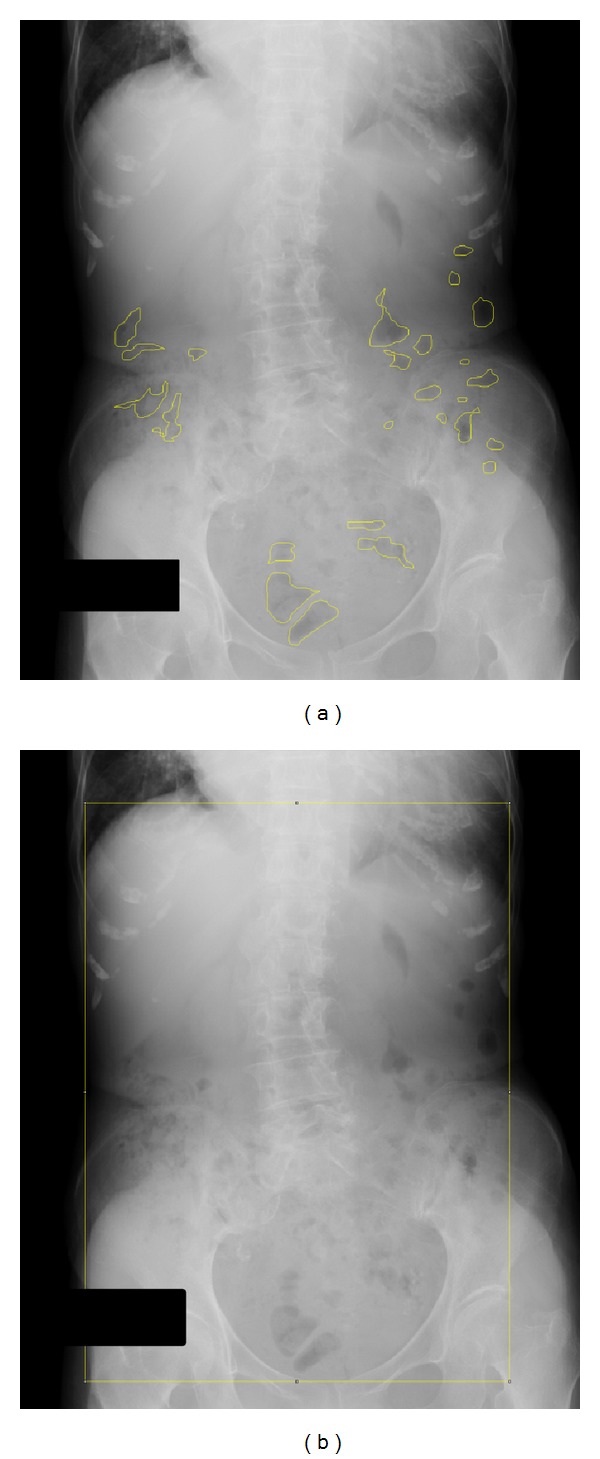
Estimation of gas volume score (GVS). Plain abdominal radiographs obtained from fasting subjects were converted to digital data. The data were read using ImageJ, an image analysis program, and intestinal gas was traced using the program. (a) Tracing image and pixel count of the gas was 3,533 in this patient. (b) The window of abdominal area. The rectangular area was measured as the area between the inferior right side margin of the diaphragm, the inner costal margin, and the superior border of the pubic symphysis. The pixel count of the rectangular area was calculated as 92,968 in (b). GVS was calculated as (a)/(b)%; therefore, the GVS of this image is “3,533/92,968 = 0.038(3.8%).”

Plain abdominal radiographs of fasting patients in a supine position were obtained before and after the trial period. The gas volume score (GVS) was calculated by Koide's method [35] using ImageJ [36] (Figure 1).

#### 2.6.4. Blood Sampling

General blood counts and biochemistry tests were performed in fasting patients before and after the trial period to assess potential adverse effects. Blood sample portions were stored in EDTA-2Na tubes. Samples were centrifuged (3000 rev/10 min), and 0.5 mL of plasma was collected and stored at −20°C. The concentration of plasma CGRP was quantified using the Human CGRP Elisa Kit (MyBioSource, Inc., San Diego, USA) tested by SRL, Inc., Tokyo, Japan.

#### 2.6.5. Statistical Analysis

Statistical analysis was performed using SPSS software (ver. 16, SPSS Japan Inc., Tokyo, Japan). Baseline comparisons of group differences were conducted using the independent samples* t*-test for continuous variables and the chi-square test for categorical variables. Measurement of the mean and standard deviation (SD) was performed at baseline and at the endpoint for all parameters. Comparisons between the DKT and control groups were performed by two-way analysis of variance (ANOVA). Changes within groups before and after the trial period were compared using the paired* t*-test when the intergroup difference was significant. Correlation between age and the CSS points was analyzed by coefficient of product-moment correlation (Pearson correlation coefficient).* P* values <0.05 were considered significant.

## 3. Results

From September 2012 to December 2013, 34 subjects (17 men and 17 women; mean age: 78.1 ± 11.6 years) at 6 hospitals participated in the study. Patients were randomly assigned to 2 groups (control group or DKT group). The demographic characteristics, CSS, and GVS of each group at baseline are shown in Table 1. There was no significant difference between groups in characteristics, the way of nutrition intake, CSS, or GVS at baseline.

### 3.1. Changes in Clinical Constipation Scores

All 34 subjects completed the CCS questionnaire before and after the observation period, and results are summarized in Table 2. There was no significant correlation between age and the CSS points on the baseline (*n* = 34) (*r* = 0.12, *P* = 0.49). Significant differences in the CSS scores were observed between the 2 groups (two-way ANOVA, *P* < 0.01). In the DKT group, the CSS scores significantly improved from 8.0 ± 3.1 to 6.0 ± 3.1 points (paired* t*-test, *P* < 0.01). There was no significant correlation between age and the changes of the CSS scores for subjects in DKT group (*n* = 17) (*r* = −0.16, *P* = 0.53). The control group did not show any significant improvement (Table 2). CSS subcategory findings are summarized for both groups in Table 3. Among the CSS subcategories, there were significant differences between the DKT and control groups using two-way ANOVA for the following questions: Q1 (frequency of bowel movements; *P* < 0.01), Q3 (feeling of incomplete evacuation; *P* = 0.03), and Q6 (need for drugs/enema/disimpaction; *P* = 0.02). In the DKT group, the constipation scores significantly decreased over the trial period for Q1 (*P* < 0.01), Q3 (*P* = 0.049), and Q6 (*P* = 0.03). The control group, however, did not show any significant changes (Table 3). Overall, the average change of 1 point in the score for Q1 means an improvement in defecation frequency from “once per week” to “2 times per week” or “less than once per week” to “once per week” in the clinical setting. The average change of 0.4 points in the scores for Q3 and Q6 means that digital assistance or enemas were no longer necessary for approximately 30% of the patients in the DKT group.

### 3.2. Changes in Gas Volume Score

**Figure 2:**
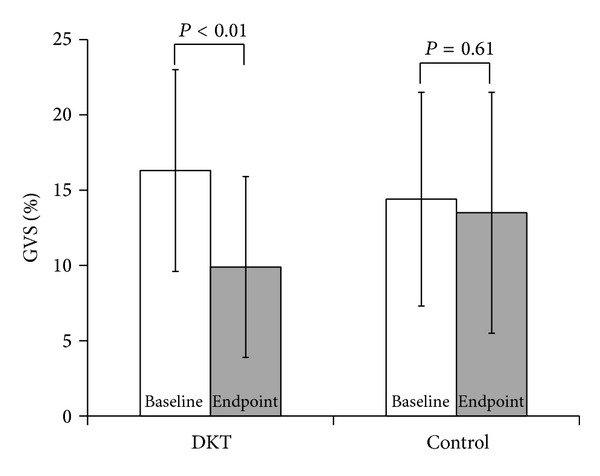
Changes in the gas volume score (GVS). Two-way ANOVA showed a significant difference between the groups (*P* = 0.03). In the DKT group, the GVS significantly improved from 16.3 ± 6.7% to 9.9 ± 6.0% (paired* t*-test; *P* < 0.01), and in the control group it changed from 14.4 ± 7.1% to 13.5 ± 8.0% with no significance (paired* t*-test; *P* = 0.61).

**Figure 3:**
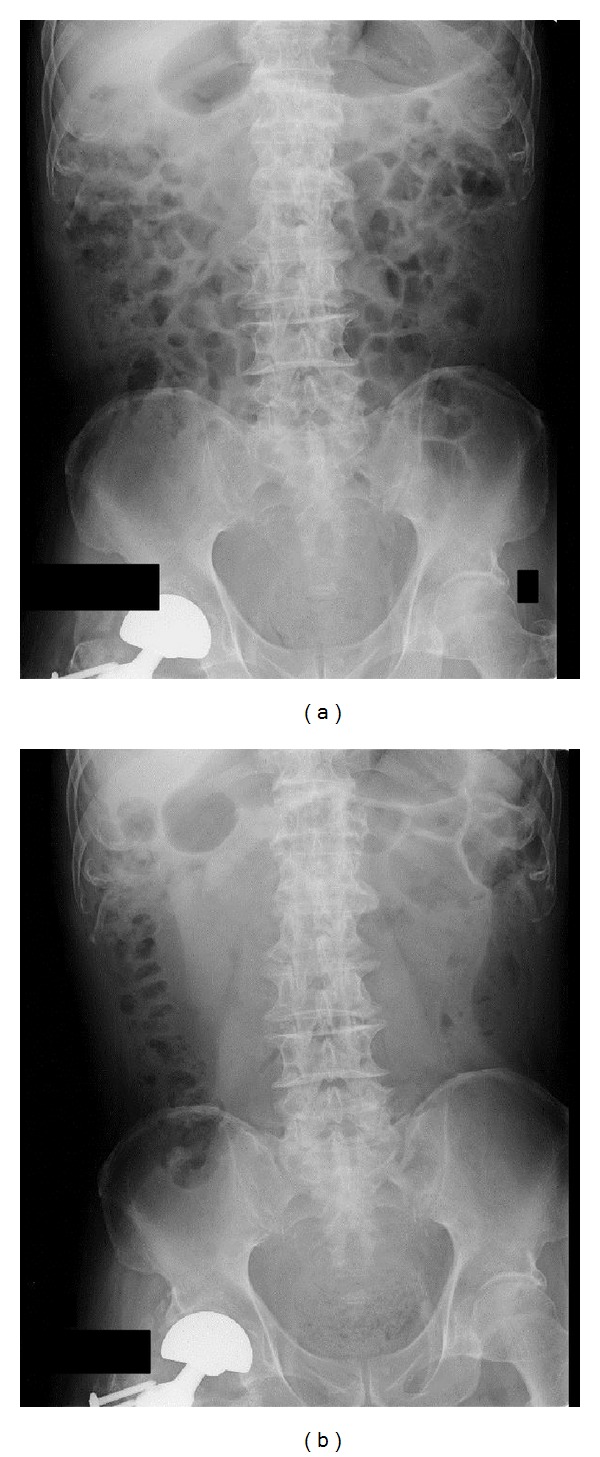
(a) Plain abdominal radiograph of an 86-year-old man prior to Daikenchuto administration. The gas volume score (GVS) was calculated as 26.0%. (b) Plain abdominal radiograph of an 86-year-old man after 4 weeks of Daikenchuto administration. The gas volume score (GVS) was calculated as 12.3%.

Figure 2 summarizes changes in the GVS before and after the observation period for both groups. There was a significant difference between the 2 groups (two-way ANOVA; *P* = 0.03), and the intragroup comparison revealed a significant decrease in the DKT group from 16.3 ± 6.7% to 9.9 ± 6.0% (*P* < 0.01) while the control group did not show any significant changes (*P* = 0.61). Representative abdominal radiographs of a patient before and after DKT administration show reduced intestinal gas volume (Figures 3(a) and 3(b)). In this case, DKT administration reduced the GVS from 26.0% to 12.3%.

### 3.3. Changes in Plasma Calcitonin Gene-Related Peptide Concentrations

In the DKT group, the initial and final CGRP concentrations were 409 ± 482 pg/mL and 452 ± 574 pg/mL, respectively. In the control group, the initial and final values were 270 ± 172 pg/mL and 251 ± 118 pg/mL, respectively. There was no significant difference between the 2 groups in plasma CGRP (two-way ANOVA; *P* = 0.08).

### 3.4. Adverse Effects

Notable adverse effects, such as itching, gastrointestinal symptoms, other subjective symptoms, and abnormalities in blood counts and blood biochemistry, were not observed during and after DKT administration.

## 4. Discussion

This study shows that DKT in addition to conventional therapy for functional constipation significantly improved the CSS scores and significantly reduced the GVS in poststroke patients. The incidence of adverse effects associated with DKT extract, such as gastrointestinal discomfort and liver dysfunction, has been reported as 1.9% in prior studies [37], but no adverse effects were observed during the 4-week treatment period in the present study. Functional constipation has a complex pathophysiology, and intestinal function is controlled by the autonomic nervous system; consequently, therapeutic protocols are limited in poststroke patients [38, 39]. Several clinical studies of DKT therapy for constipation have been reported, but almost all of these were limited to healthy subjects or were case series. The present study was a prospective randomized controlled trial for functional constipation in patients with stroke-related morbidity and therefore could show stronger evidence than previous reports of the clinical effects of DKT.

In a prior clinical study, it was reported that DKT extract improved colorectal function in patients diagnosed with Parkinson's disease [40]. Another study reported that administration of DKT to patients with chronic intractable constipation improved abdominal bloating and pain symptoms [8]. The present study similarly found improvement in clinical constipation scores and GVS. Numerous studies have investigated the active ingredients and mechanisms underlying the improved intestinal motility. Intestinal contraction may be induced by DKT through the cholinergic nervous system via serotonin receptors [13, 27, 28], motilin activity [23, 24], and the transient receptor potential vanilloid type 1 channel [11, 41]. Satoh et al. reported that* Zanthoxylum* fruit and maltose, ingredients in DKT, improved delayed propulsion in the small intestine.* Zanthoxylum* fruit also improved delayed propulsion in the distal colon. Endogenous cholecystokinin secretion resulting from maltose administration may play a role in the effect of DKT [42]. These reports describe the possible mechanisms through which DKT promotes intestinal movement and explain some aspects of the improvement in the CSS scores and the reduction of GVS noted in our study.

Some studies reported that DKT extract increased CGRP in healthy subjects [21, 25]. In another report, DKT did not change CGRP levels in patients with constipation secondary to palliative morphine therapy for cancer [24]. In the present study, changes in CGRP did not reach statistical significance. Several mechanisms may explain this lack of change in CGRP levels in the DKT group. Plasma CGRP is notably unstable [43]. An elevation following DKT administration may have been obscured by factors such as testing procedures, individual differences, daily fluctuations, and day-to-day variations. Furthermore, although some studies confirmed elevated CGRP immediately after DKT administration [21, 25], the CGRP level may be too unstable to be used as a target factor for evaluating the effects of DKT. DKT is thought to affect the promotion of intestinal motility and intestinal blood flow. Increase in intestinal blood flow is believed to be mediated through adrenomedullin and CGRP or through the transient receptor potential ankyrin 1 channel [16, 29, 30]. The mechanisms promoting intestinal motility and blood flow have complex interactions, which may be altered further by disease pathology, environment, and individual differences. The present results of improved constipation following DKT administration are overall consistent with the findings of prior studies, despite the lack of significant change in CGRP levels.

### 4.1. Limitations

The small sample size is the first limitation of the present study. The CGRP level tended to differ between the groups (ANOVA, *P* = 0.08); a larger sample size could determine the significance of this difference. In addition, participants were limited to hospitalized patients; therefore, patients who were hemiplegic, yet stable enough to receive outpatient care, were not included. As a result, the population was skewed toward patients with low activities of daily living. Third, there are no objective parameters for abdominal coldness at present. Ultrasound assessment of blood flow in the superior mesenteric artery was nearly impossible in poststroke patients with constipation owing to the presence of intestinal gas. Finally, the placebo effect of oral administration cannot be overlooked. A randomized double-blind comparative study using a placebo would be ideal and would eliminate the placebo effect. DKT includes 4 crude herbs and has a sweet and hot flavor. It will be difficult to produce a placebo without bioactivity that has a smell and flavor similar to DKT. Accordingly, the present study did not use a placebo control but rather compared the effects of DKT administration plus conventional treatment to conventional treatment alone.

## 5. Conclusions

Administration of DKT extract in conjunction with conventional therapy to treat functional constipation in poststroke patients improved clinical constipation scores and reduced intestinal gas volume. Results of this study show that DKT is effective for defecation control in poststroke patients.

## Figures and Tables

**Table 1:** Baseline population demographics of DKT and control groups.

	Group		P*
	DKT^a^	Control	
*N*	17	17	
Sex			0.73
Female	9	8	
Male	8	9	
Age (y)	77.5 ± 11.9	78.7 ± 12.1	0.78
Height (cm)	156.3 ± 12.1	154.1 ± 9.3	0.56
Body weight (kg)	48.4 ± 10.2	48.3 ± 9.4	0.99
Diagnoses, *N*			0.31
Brain infarction	10	14	
Cerebral hemorrhage	4	2	
Subarachnoid hemorrhage	3	1	
Illness duration (y)	7.8 ± 6.1	4.8 ± 4.2	0.15
Barthel Index	2.1 ± 3.1	1.2 ± 2.8	0.39
The way of nutritional intake			0.14
Orally	5	1	
Through nasogastric tube	2	5	
Through gastrostomy tube	10	11	
CSS total^b^ (points)	8.0 ± 3.1	8.1 ± 3.7	0.96
CGRP (pg/mL)	408 ± 482	262 ± 170	0.25
GVS (%)	16.3 ± 6.7	14.4 ± 7.8	0.44

^a^DKT, Daikenchuto; CSS, constipation scoring system; CGRP, calcitonin gene-related peptide; GVS, gas volume score.

^b^CSS total: not including point of Q5.

*Significance designated at *P* < 0.05.

**Table 2:** Clinical constipation scores in both groups at baseline and endpoint.

	DKT^a^ group (*N* = 17)		Intragroup difference	Control group (*N* = 17)		Intragroup difference	Intergroup difference
	Baseline	Endpoint^b^	P*	Baseline	Endpoint	*P*	*P*
CSS total^c^ (points)	8.0 ± 3.1	6.0 ± 3.1	<0.01	8.1 ± 3.7	8.2 ± 3.7	0.33	<0.01

^a^DKT, Daikenchuto; CSS, constipation scoring system.

^b^Endpoint: after the 4-week trial period.

^c^CSS total: not including point of Q5.

*Significance designated at *P* < 0.05.

**Table 3:** Constipation scoring system (CSS) subcategory scores in both groups at baseline and endpoint.

	DKT^a^ group (*N* = 17)		Intragroup difference	Control group (*N* = 17)		Intragroup difference	Intergroup difference
	Baseline	Endpoint^b^	P*	Baseline	Endpoint	*P*	*P*
Q1 (points)	2.2 ± 1.5	1.2 ± 1.4	<0.01	2.1 ± 1.4	2.1 ± 1.5	0.33	<0.01
Q2 (points)	0.5 ± 0.9	0.3 ± 0.7	—	0.6 ± 0.9	0.6 ± 0.9	—	0.07
Q3 (points)	1.2 ± 1.2	0.8 ± 1.0	0.049	1.5 ± 1.3	1.6 ± 1.4	0.33	0.03
Q4 (points)	0.4 ± 0.8	0.4 ± 0.7	—	0.7 ± 0.9	0.7 ± 0.9	—	0.33
Q5 (points)	—	—	—	—	—	—	—
Q6 (points)	1.8 ± 0.5	1.4 ± 0.8	0.03	1.7 ± 0.7	1.7 ± 0.7	1.00	0.02
Q7 (points)	0.1 ± 0.2	0.1 ± 0.2	—	0.1 ± 0.2	0.1 ± 0.2	—	1.00
Q8 (points)	1.9 ± 1.1	1.9 ± 1.1	—	1.5 ± 0.9	1.5 ± 0.9	—	1.00

Intragroup difference was calculated using the paired *t*-test only when the intergroup difference was significant.

^a^DKT, Daikenchuto.

^b^Endpoint: after the 4-week trial period.

*Significance designated at *P* < 0.05.

## Appendix

### Constipation Scoring System (CSS) [34]

Minimum score, 0; Maximum score, 30, the numbering starting from zero represents the scores.

(1) Frequency of bowel movements
  (0) 1-2 times per 1-2 days
  (1) 2 times per week
  (2) Once per week
  (3) Less than once per week
  (4) Less than once per month
(2) Difficulty: painful evacuation effort
(3) Completeness: feeling incomplete evacuation
(4) Pain: abdominal pain
  (0) Never
  (1) Rarely
  (2) Sometimes
  (3) Usually
  (4) Always
(5) Time: minutes in lavatory per attempt
  (0) Less than 5
  (1) 5−10
  (2) 10−20
  (3) 20−30
  (4) More than 30
(6) Assistance: type of assistance
  (0) Without assistance
  (1) Stimulative laxatives
  (2) Digital assistance or enema
(7) Failure: unsuccessful attempts for evacuation per 24 hours
  (0) Never
  (1) 1–3
  (2) 3–6
  (3) 6–9
  (4) More than 9
(8) History: duration of constipation (yr)
  (0) 0
  (1) 1–5
  (2) 5–10
  (3) 10–20
  (4) More than 20
